# Rate of candidiasis among HIV-infected children in Spain in the era of highly active antiretroviral therapy (1997–2008)

**DOI:** 10.1186/1471-2334-13-115

**Published:** 2013-03-04

**Authors:** Alejandro Álvaro-Meca, Julia Jensen, Dariela Micheloud, Asunción Díaz, Dolores Gurbindo, Salvador Resino

**Affiliations:** 1 Unidad de Medicina Preventiva y Salud Pública, Universidad Rey Juan Carlos, Alcorcón, Madrid, Spain; 2 Servicio de Pediatría, Hospital Infanta Cristina, Madrid, Parla, Spain; 3 Servicio de Medicina Interna .Hospital General Universitario Gregorio Marañon, Madrid, Spain; 4 Centro Nacional de Microbiología, Instituto de Salud Carlos III, Carretera Majadahonda- Pozuelo, Km 2.2, Madrid, Majadahonda 28220, Spain; 5 Centro Nacional de Epidemiología, Instituto de Salud Carlos III, Madrid, Spain; 6 Servicio de Pediatría, Hospital General Universitario Gregorio Marañon, Madrid, Spain

**Keywords:** AIDS, Candidiasis, HAART, Infection, Pediatric, Epidemiology

## Abstract

**Background:**

Candidiasis is the most common opportunistic infection seen in human immunodeficiency virus (HIV)-infected individuals. The aim of our study was to estimate the candidiasis rate and evaluate its trend in HIV-infected children in Spain during the era of highly active antiretroviral therapy (HAART) compared to HIV-uninfected children.

**Methods:**

We carried out a retrospective study. Data were obtained from the records of the Minimum Basic Data Set from hospitals in Spain. All HIV-infected children were under 17 years of age, and a group of HIV-uninfected children with hospital admissions matching the study group by gender and age were randomly selected. The follow-up period (1997–2008) was divided into three calendar periods: a) From 1997 to 1999 for early-period HAART; b) from 2000 to 2002 for mid-period HAART; and c) from 2003 to 2008 for late-period HAART.

**Results:**

Among children with hospital admissions, HIV-infected children had much higher values than HIV-uninfected children during each of the three calendar periods for overall candidiasis rates (150.0 versus 6.1 events per 1,000 child hospital admissions/year (p < 0.001), 90.3 versus 3.1 (p < 0.001), and 79.3 versus 10.7 (p < 0.001), respectively) and for non-invasive Candida mycosis (ICM) rates (118.5 versus 3.8 (p < 0.001), 85.3 versus 2.3 (p < 0.001), and 80.6 versus 6.0 (p < 0.001), respectively). In addition, HIV-infected children also had higher values of ICM rates than HIV-uninfected children, except during the last calendar period when no significant difference was found (32.4 versus 1.2 (p < 0.001), 11.6 versus 0.4 (p < 0.001), and 4.6 versus 2.3 (p = 0.387), respectively). For all children living with HIV/AIDS, the overall candidiasis rate (events per 1,000 HIV-infected children/year) decreased from 1997–1999 to 2000–2002 (18.8 to 10.6; p < 0.001) and from 2000–2002 to 2003–2008 (10.6 to 5.7; p = 0.060). Within each category of candidiasis, both non-ICM and ICM rates experienced significant decreases from 1997–1999 to 2003–2008 (15.9 to 5.7 (p < 0.001) and 4.1 to 0.3 (p < 0.001), respectively).

**Conclusions:**

Although the candidiasis rate still remains higher than in the general population (from 1997 to 2008), candidiasis diagnoses have decreased among HIV-infected children throughout the HAART era, and it has ceased to be a major health problem among children with HIV infection.

## Background

In developed countries, the number of human immunodeficiency virus (HIV)-infected children receiving highly active antiretroviral therapy (HAART) has increased since 1996. During its earliest years, HAART had important limitations such as only a few approved pediatric formulations of protease inhibitor (PI), difficulties with administration and low adherence, as well as issues with unsuitable formulations and inadequate pharmacokinetics [1-3]. The introduction of new drugs and formulations after 1999 [4,5] extended the use of HAART to a larger number of children [6]. And after 2002, new HAART regimens allowed for increased adherence and effectiveness [7].

In the pre-HAART era, opportunistic infections were the primary cause of death in HIV-infected children [8,9]. Current HAART regimens work by achieving maximal viral load suppression and increasing CD4+ T-cell (CD4+) counts [10], and have resulted in a substantial and dramatic decrease in acquired immunodeficiency syndrome (AIDS)-related opportunistic infections, morbidity, hospitalizations and deaths in HIV-infected children [8,11-15].

Candidiasis is the most common opportunistic infection seen in HIV-infected children, particularly in HIV-infected children with persistently low CD4+ counts [16]. Localized disease caused by Candida is characterized by limited tissue invasion in the skin or mucosa. Once the organism penetrates the mucosal surface and widespread hematogenous dissemination occurs, invasive Candida mycosis (ICM) ensues [17]. ICM usually occurs in critical situations with advanced stages of immune-suppression [16], and it has proved to be a significant cause of morbidity in HIV-infected patients during the course of hospitalization [17]. However, the impact of HAART has resulted in a decline in the incidence of candidiasis in this population [11,14,18]. Candida-specific immunity is one of the earliest to recover in response to HAART, which is consistent with increases in CD4+ counts and the disappearance of clinical signs in response to HAART [19,20].

The aim of our study was to estimate the candidiasis rate and evaluate its trend in HIV-infected children in Spain during the HAART era compared to HIV-uninfected children.

## Methods

### Study population

**Figure 1:**
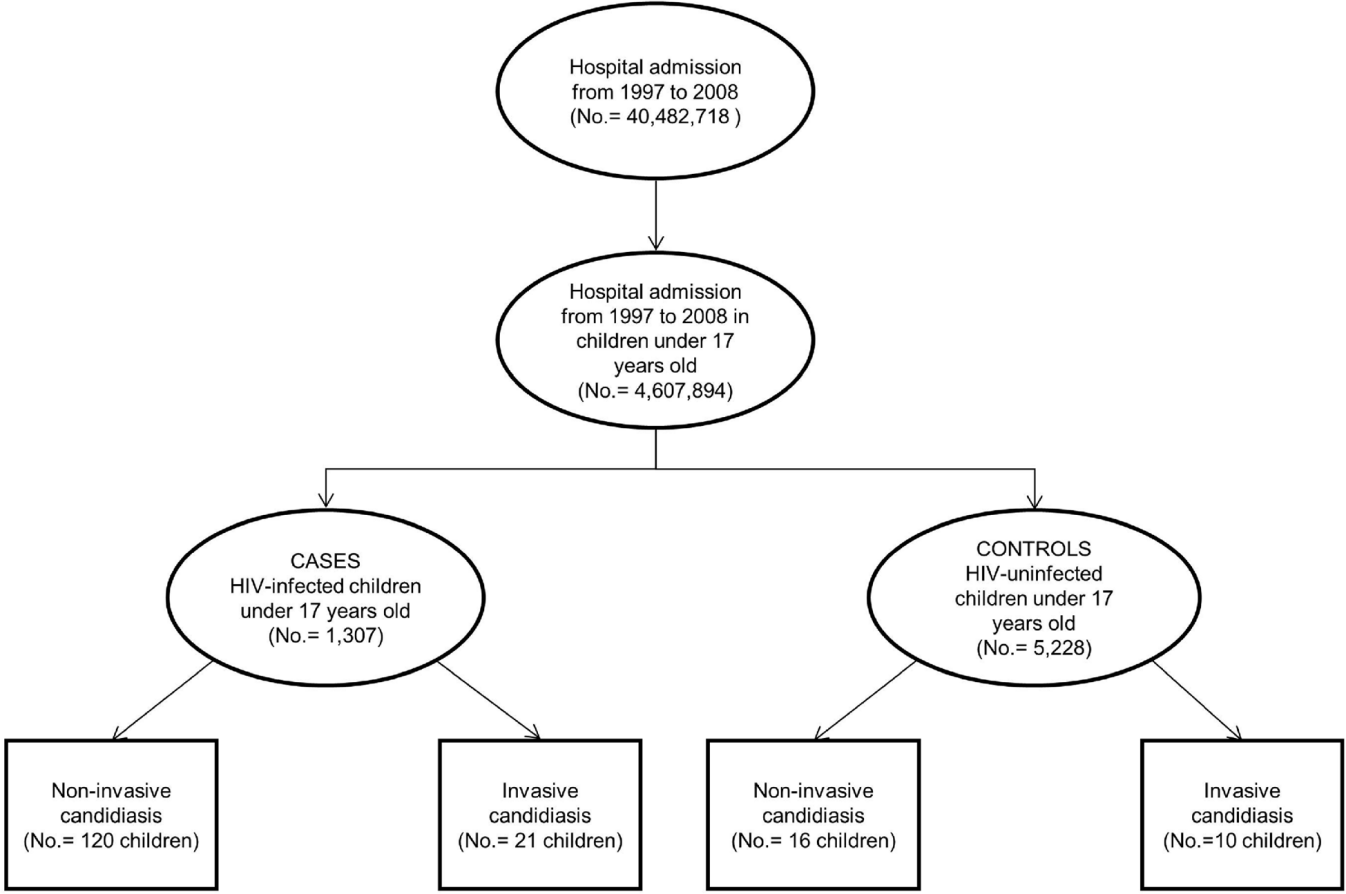
Flow-chart for the selection of HIV-infected children (cases) and HIV-uninfected children (controls) under 17 years old and candidiasis diagnosis in Spain from 1997 to 2008.

We carried out a retrospective study from 1997 to 2008 among all HIV-infected children under 17 years of age with a hospital admission in Spain (Figure 1) [21]. In addition, we selected a control group composed of 4 HIV-uninfected children for each HIV-infected child studied. This control group (selective ‘cohort’ which has not been exposed to HIV or HAART) was randomly selected from all HIV-uninfected children under 17 with hospital admissions and matched by gender and age.

Data were obtained from the records of the Minimum Basic Data Set (MBDS) of hospitals in Spain, as we previously described [21]. The MBDS is a database that captures 97.7% of all public hospital admissions and 25% of all private hospital admissions in Spain. We did not have data permitting us to estimate the proportion of HIV-infected children in Spain who received care at a private hospital. However, all HIV-infected children are treated through the public health system in Spain and, thus, would be likely to receive their care at public hospitals. The MBDS system showed no significant variations during the whole study period (1997–2008).

Data were treated with full confidentiality, according to Spanish legislation. Patient identifiers were deleted before the database was provided to the authors in order to keep strict patient confidentiality. It is not possible to identify patients at individual levels either in this paper or in the database. Given the anonymous and mandatory nature of the data, informed consent was neither required nor necessary. The Spanish Ministry of Health evaluated the protocol of our investigation and considered that it met all ethical aspects according to Spanish legislation so provided us with the database. The study was approved by the Research Ethic Committee of the Instituto de Salud Carlos III (ISCIII).

The MBDS provides the encrypted patient identification number, sex, date of birth, dates of hospital admission and discharge, medical institutions providing the services, the *International Classification of Diseases, 9th ed, Clinical Modification* (ICD-9-MC) codes of diagnoses and procedures, and outcome at discharge. In this study, HIV infection was assigned to patients who had an ICD-9-CM code of 042 (HIV disease) or V08 (Asymptomatic HIV infection status).

### Exposure variables

We analyzed two kinds of exposure variables:

i.) HIV infection: we analyzed two study groups according to HIV status: HIV-uninfected children and HIV-infected children.

ii.) HAART, the standard treatment for HIV-infected children: in this study, we divided the follow-up period from 1997 to 2008 into three subperiods or calendar periods, according to the widespread use of HAART in children [21]: a) from 1997 to 1999 (1997–1999) for early-period HAART, b) from 2000 to 2002 (2000–2002) for mid-period HAART, and c) from 2003 to 2008 (2003–2008) for late-period HAART.

### Outcome variables

The index episode was defined as the occurrence of a hospital discharge with candidiasis diagnosis via ICD-9 codes:

i.) Non-ICM: candidiasis of mouth (112.0), candidiasis of vulva and vagina (112.1), candidiasis of other urogenital sites (112.2), candidiasis of skin and nails (112.3), candidal otitis external (112.82), candidal esophagitis (112.84).

ii.) ICM: candidiasis of lung (112.4), disseminated candidiasis (112.5), candidal endocarditis (112.81), candidal meningitis (112.83), candidal enteritis (112.85), other candidiasis of other specified sites (112.89), candidiasis of unspecified site (112.9), neonatal Candida infection (771.7).

Hospitalization was defined as a discharge record in the MBDS, and children who were readmitted with candidiasis in the same hospital and in the same calendar year were counted as new diagnoses of candidiasis.

### Estimation of the number of children living with HIV/AIDS in Spain from 1997 to 2008

The estimation of the number of children living with HIV/AIDS in Spain was made from two official registries of HIV-infected children (see Additional file 1), as previously described [21]:

i. The number of HIV-infected children in the Madrid cohort, which was supplied by the Madrid Cohort of HIV Children (The Madrid HIV Paediatric Infection Collaborative Study Group).

ii. The number of HIV-infected children with a diagnosis of AIDS in Spain (AIDS-S), which was supplied by the Spanish National AIDS Register (National Centre for Epidemiology, Instituto de Salud Carlos III,).

### Statistical analysis

We calculated the rate or the number of events per 1,000 children-year, for overall and specific candidiasis diagnosis, according to each calendar period. The numerator was the number of children and the number of candidiasis diagnoses among HIV-infected children within each period (whole follow-up or calendar period). The denominator was different according to the type of rate calculated: a) for the events per 1,000 children with hospital admission-year, we used number of HIV-uninfected children or HIV-infected children with a hospital discharge within the follow-up period (CMBD data); b) For the events per 1,000 HIV-infected children-year, we used the number of children living with HIV/AIDS in Spain from 1997 to 2008 (see Additional file 1).

Candidiasis rates were compared using Poisson regression. Statistical analysis was performed using the R version 2.15.0 statistical package (GNU General Public License; http://www.r-project.org/). All tests were two-tailed with p-values <0.05 considered significant.

## Results

### Study population

Figure 1 shows the number of HIV-infected children and HIV-uninfected children included in this study. We included 1307 HIV-infected children with at least one hospitalization episode. Of all these, 141 children had 149 candidiasis diagnoses, including 120 children with 128 non-ICM diagnoses and 21 children with ICM diagnoses. In addition, we included a control group of 5228 randomly selected HIV-uninfected children with hospital admissions matched by gender and age. Within the control group, 26 children had candidiasis diagnoses, including 16 children with non-ICM diagnoses and 10 children with ICM diagnoses.

Table 1 shows the epidemiological and clinical characteristics of children with candidiasis diagnoses among the cases (HIV-infected children) and controls (HIV-uninfected children) included in this study. When we analyzed within cases and controls, HIV-uninfected children with ICM diagnoses were older (p = 0.008), while HIV-infected children with ICM diagnoses had longer hospital stays (p = 0.049) than children with non-ICM diagnoses. Next, we performed the analysis among diagnostics groups (All candidiasis, non-ICM, and ICM). Overall, the percentage of males was similar among groups, but HIV-infected children were older (p = 0.016) and had longer hospital stays (p = 0.006) than HIV-uninfected children. These trends were maintained within non-ICM group. However, HIV-infected children with ICM diagnosis had higher values of percentage of males (p = 0.041) and length of stay (p < 0.001), but they had the lowest values of age (p < 0.001).

**Table 1:** Epidemiological and clinical characteristics of HIV-infected and HIV-uninfected children with candidiasis diagnoses from 1997 to 2008

	**HIV-uninfected children**				**HIV-infected children**						
	**All candidiasis**	**Non-ICM**	**ICM**	^**(a)**^**p-value**	**All candidiasis**	**Non-ICM**	**ICM**	^**(a)**^**p-value**	^**(b)**^**p-value**	^**(c)**^**p-value**	^**(d)**^**p-value**
**N**	26	16	10		141	120	21				
**Males **^**#**^	10 (38.5)	8 (50.0)	2 (20.0)	0.264	70 (49.6)	56 (46.7)	14 (66.7)	0.145	0.403	1	**0.041**
**Age (years)** *	0.5 (0; 8.7)	0 (0; 3.2)	9 (1.5; 11.0)	**0.008**	7 (1; 11)	7 (1; 11)	4 (2; 11)	0.921	**0.016**	**<0.001**	**<0.001**
**Length of stay (days)** *	6.5 (3.2; 10.5)	5.5 (3.0; 7.5)	8.5 (7.0; 22.0)	0.125	13 (7; 22)	12 (7; 22)	18 (9; 42)	**0.049**	**0.006**	**<0.001**	**<0.001**

Values expressed as: (^**#**^**)** Absolute number (percentage); (^*^) Median (percentile 25; percentile 75).

Abbreviations: ICM, invasive candidiasis mycosis; HIV, Human immunodeficiency virus.

Significance level (p-values): (a), Non-ICM vs. ICM within a group; (b), all candidiasis between groups, (c); Non-ICM between groups, (d), ICM between groups.

Statistically significant differences are shown in bold.

### Candidiasis rates among children with hospital admissions

Among children with hospital admissions, HIV-infected children had a higher overall rate of candidiasis (events per 1,000 children with hospital admission/year) than HIV-uninfected children (107.0 versus 4.9; p < 0.001) (Table 2). By type of candidiasis diagnosis, HIV-infected children had higher rates of both non-ICM and ICM diagnoses than HIV-uninfected children (91.8 versus 3.1 (p < 0.001) and 16.1 versus 1.9 (p < 0.001), respectively) (Table 2). HIV-infected children also had higher rates of non-ICM than ICM diagnoses (p < 0.001) (Table 2). The most frequent diagnosis in both groups of children (HIV-uninfected and HIV-infected children) was candidiasis of mouth.

**Table 2:** Summary of the number and rates of children with candidias per 1,000 children with hospital admission/year (bold font) and specific candidiasis diagnoses per 1,000 children with hospital admission/year (non-bold font) in HIV-infected children and HIV-uninfected children in Spain from 1997 to 2008

		**HIV-uninfected children**		**HIV-infected children**	
**Description**	**ICD-9 codex**	**No.**	**Rate (95% CI)**	**No.**	**Rate (95% CI)**
**All candidiasis**	112.XX	26	5.0 (3.1; 6.9)	141	107.0 (90.1; 125.7) ^(*)^
**Non-invasive Candida mycosis (Non-ICM)**		16	3.1 (1.6; 4.6)	120	91.8 (75.4; 108.3) ^(*, †)^
Candidiasis of mouth	112.0	13	2.5 (1.1; 3.8)	104	79.6 (64.6; 94.7) ^(*)^
Candidiasis of vulva and vagina	112.1	0	-	2	1.5 (0; 3.6)
Candidiasis of other urogenital sites	112.2	0	-	4	3.1 (0.1; 6.1)
Candidiasis of skin and nails	112.3	3	0.6 (0; 1.2)	5	3.8 (0.5; 7.2) ^(*)^
Candidal otitis external	112.82	0	-	1	0.7 (0; 2.3)
Candidal esophagitis	112.84	0	-	12	9.2 (4.0; 14.4)
**Invasive Candida mycosis (ICM)**		10	1.9 (0.7, 3.1)	21	16.1 (9.2; 23.0) ^(*)^
Disseminated candidiasis	112.5	1	0.2 (0; 0.6)	3	2.3 (0; 4.9) ^(*)^
Candidal enteritis	112.85	0	-	5	3.8 (0.5; 7.2)
Other candidiasis of other specified sites	112.89	6	1.1 (0.2; 2.1)	7	5.3 (1.4; 9.3) ^(*)^
Candidiasis of unspecified site	112.9	1	0.2 (0; 0. 6)	4	3.1 (0.1; 6.1) ^(*)^
Neonatal candida infection	771.7	2	0.4 (0; 0.9)	2	1.5 (0; 3.6)

Abbreviations: ICM, invasive candidiasis mycosis; HIV, Human immunodeficiency virus; ICD-9, data are coded according to the International Classification of Diseases (9th revision, Clinical Modification [ICD-9-MC]); Rate, events per 1,000 children with hospital admission/year; 95% CI, 95% of confidence interval.

(†): Significant differences between groups of study within a candidiasis diagnosis category (p < 0.001).

(*): Significant differences between Non-ICM and ICM categories within a study group (p < 0.001).

When we divide the follow-up period into three separate calendar periods, we find that HIV-infected children had much higher values than HIV-uninfected children for overall candidiasis rates during each of the three periods (150.0 versus 6.1 (p < 0.001), 90.3 versus 3.1 (p < 0.001), and 79.3 versus 10.7 (p < 0.001), respectively) as well as for non-ICM rates (118.5 versus 3.8 (p < 0.001), 85.3 versus 2.3 (p < 0.001), and 80.6 versus 6.0 (p < 0.001), respectively) (Table 3). HIV-infected children had higher values of ICM rates than HIV-uninfected children, however during the last calendar period no significant difference was found (32.4 versus 1.2 (p < 0.001), 11.6 versus 0.4 (p < 0.001), and (4.6 versus 2.3 (p = 0.387), respectively) (Table 3). Of particular importance, the rates of overall candidiasis, non-ICM, and ICM all decreased from 1997–1999 to 2003–2008, however the differences were only significant for HIV-infected children with ICM diagnoses (p = 0.032).

**Table 3:** Summary of the number and rates of children with candidiasis per 1,000 children with hospital admission/year in HIV-infected children and HIV-uninfected children by calendar periods

	**1997-1999**		**2000-2002**		**2003-2008**	
**Description**	**No.**	**Rate (95% CI)**	**No.**	**Rate (95% CI)**	**No.**	**Rate (95% CI)**
**All candidiasis**						
HIV-uninfected children	8	6.1 (1.9; 10.3)	4	3.1 (0.1; 6.1)	14	10.7 (5.1; 16.3)
HIV-infected children	64	150.0 (106.4; 175.5)	41	90.3 (62.7; 117.9)	36	79.3 (53.4; 105.2)
p-value		**<0.001**		**<0.001**		**<0.001**
**Non-invasive Candida mycosis (Non-ICM)**						
HIV-uninfected children	5	3.8 (0.5; 7.1)	3	2.3 (0; 4.8)	8	6.0 (1.8; 10.2)
HIV-infected children	50	118.5 (85.6; 151.3)	36	85.3 (57.4; 113.2)	34	80.6 (53.5; 107.6)
p-value		**<0.001**		**<0.001**		**<0.001**
**Invasive Candida mycosis (ICM)**						
HIV-uninfected children	3	1.2 (0; 2.5)	1	0.4 (0; 1.1)	6	2.3 (0.5; 4.2)
HIV-infected children	14	32.4 (15.4; 49.4)	5	11.6 (1.4; 21.7)	2	4.6 (0; 11.1)
p-value		**<0.001**		**<0.001**		0.387

Abbreviations: ICM, invasive candidiasis mycosis; HIV, Human immunodeficiency virus; Rate, events per 1,000 children with hospital admission/yea; 95% CI, 95% of confidence interval.

Statistically significant differences are shown in bold.

### Candidiasis rates among all children living with HIV/AIDS

During the follow-up period taken as a whole, the overall rate of candidiasis in all HIV-infected children in Spain (events per 1,000 HIV-infected children/year) was 11.3 (95% of confidence interval (95% CI): 9.5; 13.1), the non-ICM rate was 9.7 (95% CI: 8.1; 11.4) and the ICM rate was 1.6 (95% CI: 0.9; 2.3).

**Figure 2:**
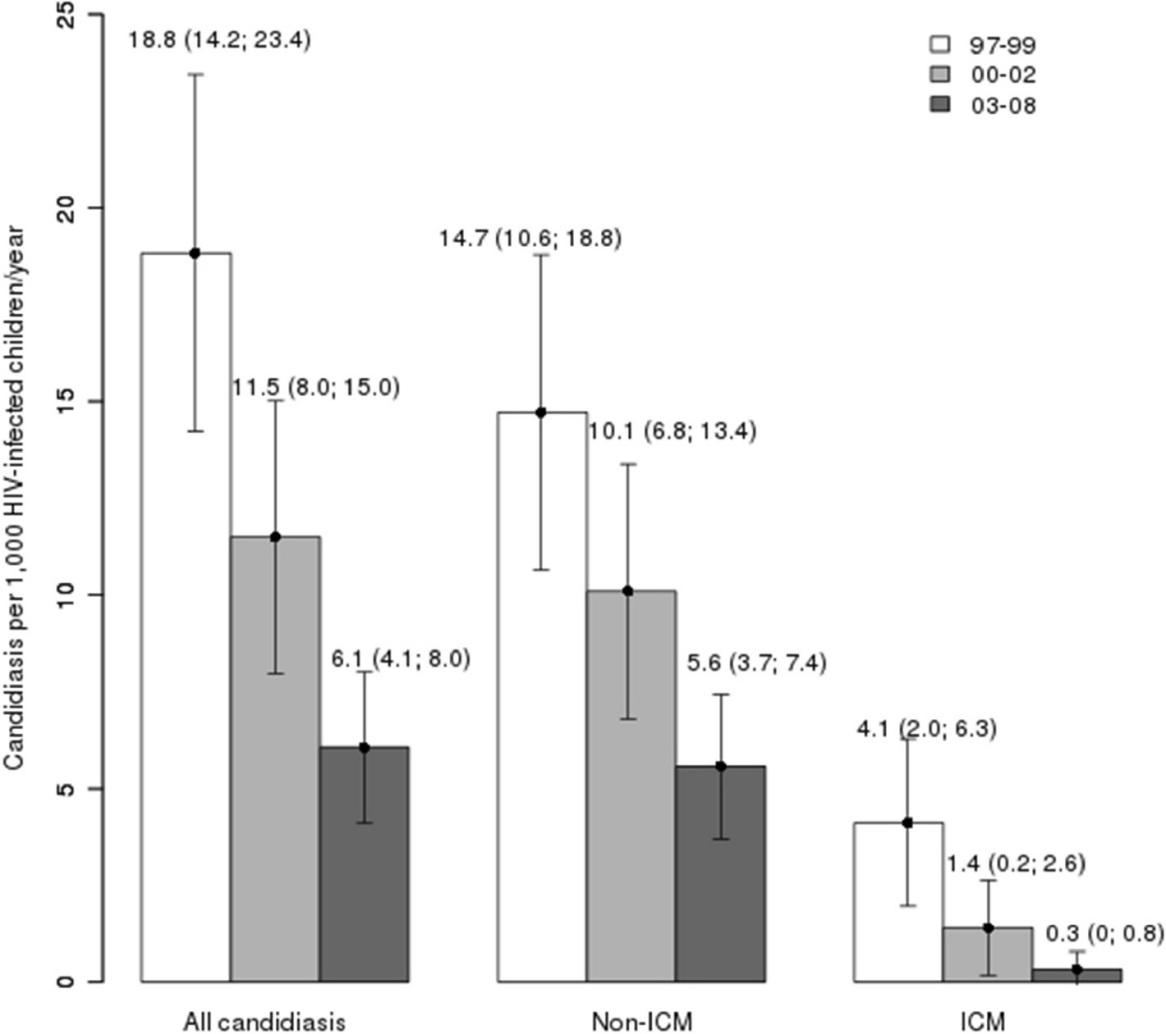
Summary of the epidemiologic trend of candidiasis (events per 1,000 HIV-infected children/year) among HIV-infected children in Spain by calendar periods.

When we divide the follow-up period into the three calendar periods, the overall candidiasis rate decreased from 1997–1999 to 2000–2002 (18.8 to 11.5; p < 0.001) and from 2000–2002 to 2003–2008 (11.5 to 6.1; p = 0.060) (Figure 2). Within each category of candidiasis, both non-ICM and ICM rates had significant decreases from 1997–1999 to 2003–2008 (14.7 to 5.6 (p < 0.001) and 4.1 to 0.3 (p < 0.001), respectively) (Figure 2). Finally, when we compared the two categories of candidiasis diagnoses, the non-ICM rates were higher than ICM rates throughout the whole follow-up and within each calendar period (Figure 2; p < 0.001).

## Discussion

Other than for oral candidiasis, there is a scarcity of literature on the rate of candidiasis (invasive or non-invasive) among HIV-infected children. This report is a retrospective study on an important part of follow-up of HIV-infected children in Spain. In our study, HIV-infected children were at increased risk of developing candidiasis (ICM and non-ICM) compared to the general population, but these rates, as with all opportunistic infections, declined with the widespread use of HAART. In addition, ICM was more infrequent than non-ICM among children of the general population as well as among children with HIV infection.

Poor cell-mediated immunity in HIV-infected children often results in opportunistic infections, especially in advanced stages of HIV infection [11]. The candida species is the most common cause of fungal infection among HIV-infected children [16]. However, HAART-derived immune reconstitution is an important protective factor against candidiasis infection [19,20]. The recovery of cellular immunity with HAART may partially reverse susceptibility to opportunistic infections when it achieves sustained undetectable levels of HIV-RNA and CD4+ levels above 500 cells/mm^3^ over a long period of time [9,11]. A high incidence of candidiasis diagnosis might be due to the lack of complete immune reconstitution and persistent CD4+ lymphopenia due to therapy failure [10]. The capacity of CD4+ recovery during long-term HAART in HIV-infected children with CD4+ levels below 5% is lower than in children with CD4+ levels from 5–15%, and restoration of the CD4+ cell percentage to a normal level is not necessarily achieved during long-term HAART [10]. Thus, continued surveillance of Candida infections may be important in order to assess the long-term effect of HAART on the occurrence of opportunistic infections in children.

Most candidiasis diagnoses were non-ICM and the rate of non-ICM diagnoses was higher in HIV-infected children than in HIV-uninfected children when children with hospital admissions were compared. However, these data should be interpreted with caution because they refer to primary and secondary diagnoses among children with hospital admissions. Since it is very rare for an HIV-uninfected child to be admitted to a hospital because of non-ICM such as oral candidiasis, the rate of non-ICM in HIV-uninfected children may be underestimated, as the database comes from hospital admissions only. Additionally, the rate of non-ICM children with hospital admissions decreased during follow-up, but no significant values were found. However, when we analyzed the data by children living with HIV/AIDS, the non-ICM rate decreased significantly in the last calendar period (2003–2008), reaching values similar to those of other pediatric populations infected with HIV [11,18,22]. This may be due to the extensive use of HAART and improved immunosurveillance, which is an important protective factor against candidiasis [19,20]. In addition, advances in HIV treatment, earlier diagnosis of infection status in exposed infants, and quantitative monitoring of virology and immunologic parameters may also have contributed to decreases in candidiasis rates. Moreover, the earlier HIV treatment starts over time, as per PENTA guidelines [23], the more non-ICM will be prevented.

Most ICM in children occur in the hospital setting, and this type of candidiasis is generally seen in severely immunocompromised persons such as cancer, transplant and AIDS patients, as well as nontrauma emergency surgery and critical ICU patients [24]. ICM is a leading cause of nosocomial mycosis with a high associated mortality [17]. In our study, HIV-infected children with ICM diagnoses had longer hospital stays than children with non-ICM diagnoses, indicating that ICM diagnoses would appear in children with more serious clinical events and suppressed immune systems [24]. Moreover, our data show that HIV-infected children were at increased risk of developing ICM, although the ICM rate decreased during the follow-up. Thus, ICM rate in the last calendar period (2003–2008) was not higher in HIV-infected children than in the general population according to our study and other reports published [17,25,26]. Therefore, it is not as important of a health problem among HIV-infected children in the last years of the HAART era.

This study had several limitations that may impact our findings. This work was a retrospective study and we had no access to patient clinical data (antiretroviral treatment regimen, duration of HAART, CD4+ count, HIV viral load, CDC stage) that might have affected our results. MBDS data are anonymous and it is impossible to identify whether a patient has been hospitalized at different hospitals within the same calendar year. This may have caused a slight overestimation of our results because we may have calculated disease exacerbations or remissions as new participants. We cannot know the total number of HIV-infected children in Spain at present, because there is no national coverage data of HIV diagnoses of children in Spain. We used an estimation of the number of HIV-infected children in Spain, which was calculated from two reliable databases (Spanish National AIDS Register and Madrid Cohort HIV Children).

## Conclusions

Although the candidiasis rate still remains higher than in the general population (from 1997 to 2008), candidiasis diagnoses decreased among HIV-infected children throughout the HAART era and it has ceased to be a major health problem among children with HIV infection in Spain.

## Abbreviations

HIV: Human immunodeficiency virus;HAART: Highly active antiretroviral therapy;ICM: Invasive candida mycosis;non-ICM: Non-invasive candida mycosis;CD4+: CD4+ T-cell;MBDS: Minimum basic data set;ICD-9-MC: International classification of diseases, 9th ed, clinical modification;AIDS: Acquired Immune Deficiency Syndrome

## Competing interests

The authors do not have any commercial or other association that might pose a conflict of interest.

## Authors’ contributions

*Conception and design:* AAM, SR. *Analysis and interpretation of data*: AAM, SR, DM, JJ. *Drafting of the manuscript:* SR, DM, JJ. *Critical revision of the manuscript:* AD, DG. *Final approval of the version:* SR. All authors read and approved the final manuscript.

## Pre-publication history

The pre-publication history for this paper can be accessed here:

http://www.biomedcentral.com/1471-2334/13/115/prepub

## Supplementary Material

Additional file 1 Estimation of the number of children living with HIV/AIDS in Spain from 1997 to 2008. Click here for file
