# Supplementary material for: Rate of candidiasis among HIV-infected children in Spain in the era of highly active antiretroviral therapy (1997–2008)
Source: BMC Infect Dis. 2013 Mar 4;13:115. doi: 10.1186/1471-2334-13-115 (PMC3599397; doi:10.1186/1471-2334-13-115)
Supplement: Additional file 1 — Estimation of the number of children living with HIV/AIDS in Spain from 1997 to 2008. [file 1471-2334-13-115-S1.docx]

**Supplemental digital content 1**. Estimation of the number of children living with HIV/AIDS in Spain from 1997 to 2008.

| **Year** | **AIDS-M *** | **Non-AIDS-M *** | **Non-AIDS-M/AIDS-M ratio** | **AIDS-S ^†^** | **Non-AIDS-S ^‡^** | **Paediatric HIV infections ^§^** |
| --- | --- | --- | --- | --- | --- | --- |
| **1997** | 101 | 163 | 1.61 | 423 | 683 | 1106 |
| **1998** | 100 | 169 | 1.69 | 417 | 705 | 1122 |
| **1999** | 100 | 178 | 1.78 | 421 | 749 | 1170 |
| **2000** | 109 | 184 | 1.69 | 424 | 716 | 1140 |
| **2001** | 102 | 196 | 1.92 | 425 | 817 | 1242 |
| **2002** | 109 | 203 | 1.86 | 414 | 771 | 1185 |
| **2003** | 103 | 209 | 2.03 | 399 | 810 | 1209 |
| **2004** | 109 | 214 | 1.96 | 379 | 744 | 1123 |
| **2005** | 106 | 216 | 2.04 | 349 | 711 | 1060 |
| **2006** | 107 | 217 | 2.03 | 327 | 663 | 990 |
| **2007** | 108 | 221 | 2.05 | 299 | 612 | 911 |
| **2008** | 107 | 220 | 2.06 | 266 | 547 | 813 |

AIDS, Acquired immunodeficiency syndrome; HIV, Human immunodeficiency virus.

(*): Number of HIV-infected children with and without a diagnosis of AIDS in the Madrid cohort (data supplied by Madrid Cohort HIV Children - The Madrid HIV Paediatric Infection Collaborative Study Group).

(^†^): Number of HIV-infected children with a diagnosis of AIDS in Spain (data supplied by Spanish National AIDS Register, National Centre for Epidemiology, ISCIII).

(^‡^): Estimated number of HIV-infected children in Spain without a diagnosis of AIDS (AIDS-S ^†^ x Non-AIDS-M/AIDS-M ratio).

(**^§^**): Estimated number of HIV-infected children in Spain (AIDS-S ^†^ + Non-AIDS-S ^‡^).
